# Persistence of antenatal anti-D immunoglobulin G at delivery in twin versus singleton pregnancies: a retrospective cohort study

**DOI:** 10.1186/s12884-026-09530-2

**Published:** 2026-06-20

**Authors:** Cemal Ünlü, Hüseyin Aksoy

**Affiliations:** 1 Department of Obstetrics and Gynecology, Special Clinic, Kayseri, Turkey; 2 Department of Obstetrics and Gynecology, Kayseri Education and Research Hospital of Medicine, Kayseri, Turkey

**Keywords:** Antenatal prophylaxis, Indirect antiglobulin test, Rhesus D incompatibility, Twin pregnancy

## Abstract

**Background:**

Anti-D immunoglobulin administered in the early third trimester to Rhesus D negative women with Rhesus D incompatibility is widely assumed to provide protection against silent fetomaternal haemorrhage until delivery. However, previous studies in singleton pregnancies demonstrated that passively acquired anti-D is frequently no longer detectable by the time of delivery. The aim of this study was to assess whether the proportion of women with detectable passively administered anti-D antibodies in maternal serum at delivery differs between twin and singleton pregnancies.

**Materials and methods:**

In this retrospective cohort study, twin and singleton pregnancies with Rhesus incompatibility that delivered at Kayseri City Hospital, Obstetrics and Gynecology Clinic between January 2020 and July 2025, in women who had not previously undergone alloimmunization and received anti-D immunoglobulin prophylaxis at 28 weeks of gestation, were compared in terms of antibody persistence determined by the indirect Coombs test of maternal serum at delivery.

**Results:**

The indirect Coombs test performed at delivery was found to be negative in 50 (70.4%) of 71 twin pregnancies and in 100 (56.5%) of 177 singleton pregnancies. This difference was found to be statistically significant.

**Conclusions:**

Twin gestations showed a lower frequency of detectable passive anti-D at delivery compared with singletons. This represents a pharmacokinetic/surrogate laboratory finding and should not be interpreted as evidence of reduced clinical effectiveness of prophylaxis. Prospective studies with serial measurements and clinical outcomes are warranted.

**Supplementary Information:**

The online version contains supplementary material available at https://doi.org/10.1186/s12884-026-09530-2.

## Introduction

Maternal Rhesus D (RhD) alloimmunization occurs when an RhD-negative woman is exposed to RhD-positive fetal erythrocytes and produces anti-D immunoglobulin G (IgG). These antibodies cross the placenta and hemolyze RhD-positive fetal cells, causing hemolytic disease of the fetus and newborn (HDFN) [1].

Routine postnatal administration of anti-D IgG substantially prevents the occurrence of HDFN and has been used since 1969 [2]. Several studies have reported a further decline in sensitisation rates after the introduction of routine antenatal anti-D prophylaxis, primarily aimed at preventing immunisation from unrecognised fetomaternal haemorrhage (FMH) in the third trimester of pregnancy [3–7]. Nevertheless, even when both antenatal and postpartum prophylaxis are provided, approximately 0.1–0.3% of susceptible women still develop anti-D antibodies [8, 9]. Serologic detectability of passive anti-D at delivery is a laboratory surrogate and does not directly measure clinical protection. One possible explanation for the persistence of these occasional cases of immunisation and HDFN, despite current prophylactic regimens, is that antenatal prophylaxis may not maintain adequate passive anti-D exposure at delivery [10].

Studies investigating the effectiveness of antenatal prophylaxis have found that most mothers have no detectable antibodies around the time of delivery [11–20]. These studies consistently showed that the decline in antibody levels to undetectable levels was independent of the fetal RhD status determined after birth [11, 13, 16]. Likewise, studies investigating the individualization of antenatal prophylaxis have not demonstrated any correlation with maternal weight, body mass index, or body surface area [11, 13, 21]. Because the passive antibodies administered for prophylaxis are of the IgG type, they are able to cross the placenta and become distributed within the fetal circulation [22]. This expanded volume of distribution likely leads to a more rapid decline in antibody levels than would be expected from the theoretical half-life estimated in non-pregnant individuals [23]. Prospective and retrospective studies addressing this issue have been conducted only in singleton pregnancies, with multiple gestations excluded [11–14, 19].

A previous study [24] demonstrated that intramuscular betamethasone has a shorter maternal half-life in twin than in singleton pregnancies. Although this observation is not direct evidence for anti-D kinetics, it supports the concept that maternal pharmacokinetics can differ in twin gestations.

Given the increased circulating volume in twin pregnancies, it can be hypothesized that the half-life of prophylactic anti-D IgG is shorter in this group, which may warrant further prospective evaluation of prophylaxis strategies in twin pregnancies. Therefore, this study aimed to investigate whether there is a difference between twin and singleton pregnancies in the detectability of passively administered anti-D antibodies in maternal serum at the time of delivery.

## Materials and methods

This is a retrospective observational cohort study conducted on 71 twin and 177 singleton pregnancies with RhD incompatibility who delivered at the Kayseri City Hospital Gynecology and Obstetrics Clinic between January 2020 and July 2025.

### Inclusion and exclusion criteria

Our study included twin pregnancies whose antibody screening tests at the first pregnancy visit and at the 28th week of pregnancy were found to be negative, who received routine anti-D immunoglobulin at the 28th week of pregnancy for antenatal prophylaxis, and who underwent antibody screening at their hospitalization for delivery, and singleton pregnancies who delivered during the same study period and were selected as controls by exact matching on completed gestational week at delivery. Pregnant women who received additional anti-D immunoglobulin due to the risk of fetomaternal bleeding, other than the routine antenatal anti-D immunoglobulin prophylaxis administered at 28 weeks of gestation, were not included in the study. Patients who were alloimmunized at the initial visit or during subsequent weeks of pregnancy, and those with incomplete documentation in their medical records regarding antibody screening or anti-D immunoglobulin administration, were also excluded from the study. Alloimmunization status was determined from blood bank records of routine antenatal antibody screening (IAT) performed at booking and repeated at ~ 28 weeks *before* administration of routine antenatal anti-D immunoglobulin. Any positive screen underwent antibody identification in the transfusion laboratory; pregnancies with immune anti-D (alloimmunization) or any other clinically significant RBC alloantibody were excluded. Of the 93 twin births occurring between January 2020 and July 2025, 71 who met all these criteria were included in the study. The same exclusion criteria were applied during the selection of the control group.

### Control group selection and matching procedure

Gestational age at delivery was handled as completed weeks. For each eligible twin pregnancy (index case), we first identified all eligible singleton pregnancies delivered in the same completed gestational week and meeting the same inclusion/exclusion criteria. From this pool, we randomly selected 2–3 singleton controls using a computer-generated random number method (sampling without replacement). If fewer than the target number of eligible singleton pregnancies were available within a given gestational-week stratum, all available eligible singletons were included. Control selection was based only on eligibility criteria and gestational week at delivery; the indirect Coombs result at delivery was not used for selection. This strategy ensured week-by-week exact matching and avoided “mean gestational age” matching using adjacent weeks. This approach represents gestational-week–stratified frequency matching (exact matching by completed gestational week), rather than individual pair matching.

### Data management

In pregnant women with negative screening results, anti-D immunoglobulin is administered for antenatal prophylaxis to patients whose antibody screening is negative after re-screening at approximately the 28th week of pregnancy. At Kayseri City Hospital, prophylaxis management for pregnancies with RhD incompatibility is carried out according to a standard protocol. In accordance with the national protocol of the Turkish Ministry of Health, all eligible RhD-incompatible pregnancies—without any dose adjustment for twin gestations—received a single standard antenatal dose of anti-D immunoglobulin at approximately 28 weeks of gestation (Rhophylac^®^, 300 µg/1500 IU; CSL Behring AG, Bern, Switzerland; intramuscular). Antibody screening is performed with an indirect antiglobulin (indirect Coombs) test at the first pregnancy visit. The small number of pregnant women identified as alloimmunized as a result of positive antibody screening undergo a different monitoring protocol. When patients are admitted to the hospital for delivery, antibody screening is repeated. For the present analysis, the ‘delivery’ IAT refers to the routine antibody screen obtained at hospital admission for delivery under the institutional protocol. If the newborn’s blood type is determined to be RhD-positive, anti-D immunoglobulin is administered to the patient within 72 h postpartum. In addition, antibody screening is performed in cases that increase the risk of fetomaternal haemorrhage during the antenatal period, and if detected negative, additional antenatal anti-D immunoglobulin prophylaxis is applied.

Demographic and clinical data were retrospectively obtained from Kayseri City Hospital’s electronic patient record system, obstetric monitoring software, and blood bank records. Age, body mass index (BMI), gestational week at delivery, indirect Coombs test result (positive/negative), and the interval between antenatal anti-D IgG administration and the indirect Coombs test obtained at admission for delivery (days) were recorded for the participants included in the study.

### Indirect antiglobulin (indirect Coombs) test analysis

Indirect antiglobulin (indirect Coombs) test **(**IAT**)** analyses were performed in serum or plasma samples on the Ortho Vision Swift analyzer using BioVue Poly (polyspecific IgG+C3d) cassettes on an automated microcolumn (gel) platform (Ortho Vision Swift, Ortho Clinical Diagnostics, USA). Fifty µL of red blood cell suspension and 40 µL of serum/plasma were added to each microcolumn well and incubated at 37 °C for 15 min, followed by centrifugation for 5 min. Reactions were automatically evaluated by the instrument’s optical system and reported using proprietary software; results were graded from 0 to 4 + according to the manufacturer’s recommendations, with ≥ 1 + considered positive. Because this is a qualitative/semi-quantitative gel IAT, the analyzer does not report an anti-D concentration (IU/mL); therefore, analytic sensitivity was operationalized using the predefined positivity threshold (reaction grade ≥ 1+). A reaction grade of 0 was considered negative.

### Statistical analysis

Continuous variables were assessed for normality using the Shapiro–Wilk test and visual inspection of Q–Q plots, and are presented as mean ± standard deviation (SD). Homogeneity of variances was assessed using Levene’s test. Between-group comparisons for continuous variables (maternal age, BMI, gestational week at delivery, and the anti-D–to-delivery antibody testing interval [days]) were performed using the independent-samples t test, with Welch’s correction applied when variances were unequal.

For categorical variables (indirect Coombs test positivity), between-group comparisons were performed using Pearson’s chi-square test; data are presented as n (%). Because singleton controls were selected by week-stratified frequency matching (exact matching on completed gestational week at delivery rather than individual pairing), we used unconditional tests and logistic regression; gestational age at delivery was controlled through exact matching. To adjust for potential confounding, multivariable binary logistic regression was performed with indirect Coombs test positivity at delivery (Coombs positive = 1) as the dependent variable and pregnancy type (twin vs. singleton) as the main predictor, adjusting for maternal age, BMI, and the anti-D–to-delivery antibody testing interval (days). Gestational age at delivery was not included as an additional covariate because it was controlled through exact matching on completed gestational week. Model fit was assessed using the Hosmer–Lemeshow goodness-of-fit test (χ²=0.001, df = 6, *p* = 1.000), providing no evidence of poor fit. Multicollinearity among covariates was evaluated using variance inflation factors, and no concerning collinearity was observed (all VIFs ≤ 1.09). Results are reported as odds ratios (ORs) with 95% confidence intervals (CIs). A two-sided p value < 0.05 was considered statistically significant. An a priori sample size calculation for a chi-square comparison of proportions (effect size w = 0.20, α = 0.05, power = 0.80) indicated a minimum total sample size of approximately 197; the final sample included 248 pregnancies. Because records with incomplete documentation were excluded a priori, analyses were conducted as complete-case analyses for all variables included in the comparisons and regression model. All statistical analyses were performed using IBM SPSS Statistics for Windows, Version 22.0 (IBM Corp., Armonk, NY, USA).

## Results

Statistically significant differences were not found between twin and singleton pregnancies in terms of BMI, gestational week at delivery, and interval between anti-D IgG and the last antenatal antibody test (days) (*P* > 0.05). However, the age difference between twin and singleton pregnancies was significant. Maternal age was higher in twin pregnancies than in singleton pregnancies (*P* < 0.05; Table 1). Similarly, a significant difference was found between twin and singleton pregnancies in terms of the positivity and negativity rates of indirect Coombs tests performed at admission for delivery. Detectable anti-D antibodies at delivery were observed at a statistically significantly lower frequency in twin pregnancies than in singleton pregnancies (29.6% vs. 43.5%, *p* = 0.043) (*P* < 0.05; Table 2). Among Coombs-positive samples, reaction strength was predominantly low-grade; the distribution of grades (1+–4+) is shown in Supplementary Table S1.

**Table 1:** Age, BMI, gestational age at delivery, and the interval between anti-D IgG administration and the last antenatal antibody test in twin and singleton pregnancies

Variable	Twin pregnancy (*n* = 71)	Singleton pregnancy (*n* = 177)	*p*	95% Confidence Interval of the difference Lower Upper		
Maternal age (years)	30.14 ± 5.51	28.55 ± 5.61	0.044	0.04	3.13	
BMI (kg/m^2^)	30.70 ± 5.94	29.83 ± 4.62	0.267	-0.68	2.43	
Gestational week at delivery	35.37 ± 1.90	35.72 ± 2.38	0.253	-0.99	0.26	
Interval between anti-D IgG and the last antenatal antibody test (days)	50.32 ± 13.80	53.44 ± 10.54	0.056	-6.32	0.08	

Values are mean ± SD. p values are from independent-samples t test; Welch’s correction was applied when Levene’s test indicated unequal variances. Confidence intervals refer to the mean difference (Twin − Singleton)

**Table 2:** Indirect Coombs test results at admission for delivery in twin and singleton pregnancies

Indirect Coombs test	Twin pregnancy *n*(%)	Singleton pregnancy *n*(%)	
Positive	21 (29.6)	77 (43.5)	
Negative	50 (70.4)	100 (56.5)	
Total	71(100)	177(100)	

Values are n (%). Between-group comparison was performed using Pearson’s chi-square test: **χ²** [1] **=** 4.11, *p* = 0.043

In multivariable logistic regression adjusting for maternal age, BMI, and interval between anti-D IgG and the last antenatal antibody test (days), twin pregnancy remained independently associated with lower odds of indirect Coombs Test positivity at delivery (adjusted OR 0.27, 95% CI 0.13–0.58; *p* = 0.001) (Table 3).

**Table 3:** Logistic regression for Indirect Coombs test positivity at delivery

Predictor	B	S.E.	Wald	*p*	Exp(B)	95% CI for Exp(B) Lower Upper	
Twin pregnancy (vs. singleton)	-1.314	0.390	11.364	0.001	0.269	0.125	0.577
Maternal age (years)	0.269	0.038	49.856	< 0.001	1.309	1.214	1.410
BMI (kg/m^2^)	0.044	0.032	1.841	0.175	1.045	0.981	1.113
Interval between anti-D IgG and the last antenatal antibody test (days)	0.004	0.014	0.068	0.795	1.004	0.977	1.031

Dependent variable: indirect Coombs positivity at delivery (1 = positive). The model was adjusted for maternal age, BMI, and the anti-D IgG–to-testing interval (days). Maternal age was included as an adjustment covariate and may proxy unmeasured factors (e.g., ART status); therefore, covariate estimates should be interpreted cautiously and not causally

## Discussion

In our study, we investigated whether the persistence of maternal serum anti-D antibodies at delivery differed between RhD incompatible twin and singleton pregnancies that had received antenatal anti-D immunoglobulin prophylaxis at 28 weeks of gestation. We found that the percentage of women with detectable anti-D antibodies at delivery was statistically significantly lower in twin pregnancies compared with singleton pregnancies. Importantly, serologic detectability of passive anti-D at delivery is a laboratory surrogate and is not a validated proxy for clinical efficacy. Therefore, these data cannot be used to draw conclusions about prevention of RhD alloimmunization or HDFN.

In our study, the mean gestational age at delivery in twin pregnancies was 35 weeks and 3 days, which is consistent with the general literature [25, 26]. The incidence of preterm birth in twin pregnancies is substantially higher than in singleton pregnancies [27, 28]. However, the overall frequency of twin pregnancies is considerably lower than that of singleton pregnancies [29]. Although preterm birth is less common in singleton pregnancies, the greater number of singleton pregnancies compared with twin pregnancies allowed us to construct a singleton control group using exact matching by completed gestational week at delivery, with random selection of controls within each week stratum to minimize selection bias. Since the interval between anti-D immunoglobulin administration and delivery is the main factor influencing anti-D antibody positivity at the time of delivery, matching pregnancies by gestational age at delivery was particularly important in the selection of the control group [11, 12, 14, 19, 23].

The high rate of undetectable passive anti-D antibodies observed in our study (56.5% of singleton pregnancies at delivery) is broadly consistent with data reported in the literature following a single 1500 IU dose of antenatal anti-D immunoglobulin administered at 28 weeks of gestation [11, 14, 16, 19]. For example, other studies utilizing similar regimens documented undetectable anti-D levels at delivery in ranges such as 40.5% to 64.4% [16, 19]. Even a prospective study employing a two-step prophylaxis regimen (two 500 IU doses) yielded a high undetectable rate of 70% in women delivering near term [11]. These findings collectively suggest that, for a substantial proportion of patients, the current standard prophylaxis regimen may not maintain measurable passive anti-D levels up to the point of delivery. However, contrasting evidence exists, with some reports indicating a significantly higher persistence of antibodies. For instance, a small study reported antibody negativity in only 11.1% of participants at 35 weeks using an identical prophylaxis dose [15], whereas another found all participants to be antibody-positive at 37 weeks after 1500 IU administration, a discrepancy that may relate to differences in the route of administration [13]. Importantly, all aforementioned data are derived exclusively from singleton pregnancies. To our knowledge, the persistence and efficacy of anti-D antibodies following antenatal prophylaxis have not yet been investigated in twin pregnancies, highlighting a crucial gap in current knowledge.

Twin and singleton pregnancies differ in physiology and care patterns beyond fetal number (e.g., plasma volume expansion, gestational weight gain, and surveillance intensity), which may confound antibody persistence. Although we matched by gestational week at delivery and adjusted for maternal age, BMI, and the anti-D IgG-to-testing interval, residual confounding cannot be excluded in this retrospective cohort.

A statistically significant difference between twin and singleton pregnancies was observed in the proportions of positive and negative maternal serum indirect Coombs test results obtained at admission for delivery. This finding is consistent with altered persistence of passive anti-D in twin pregnancies and may reflect differences in distribution and/or clearance; however, in the absence of serial measurements, pharmacokinetic inferences remain speculative. One of the principal mechanisms thought to be responsible for the elimination of IgG antibodies from the body is the phagocytosis of antigen–antibody complexes by macrophages in the spleen and liver [30, 31]. However, numerous previous studies demonstrated that this mechanism does not appear to have a major influence on the half-life of anti-D IgG in pregnant women, as the duration of antibody persistence has been reported to be independent of fetal RhD status [11, 13, 16]. Another mechanism involves the continuous endocytosis of IgG antibodies by endothelial cells, with a proportion of endocytosed IgG being rescued and returned to the circulation via the neonatal Fc receptor (FcRn), whereas the remainder undergoes lysosomal degradation [32–34]. FcRn-mediated recycling is pH-dependent: IgG binds FcRn in acidic endosomes and is released at neutral pH, thereby protecting IgG from lysosomal catabolism and contributing to its prolonged serum persistence [35]. At the maternofetal interface, FcRn expressed on syncytiotrophoblast cells mediates maternal-to-fetal IgG transcytosis, and transfer efficiency increases with advancing gestation [36].

In twin pregnancies, IgG antibodies can cross the placenta [22] and are therefore expected to distribute into the circulation of both fetuses. Increased intravascular volume and greater exposure of circulating antibodies to endothelial cells in twin pregnancies may contribute to reduced detectability/persistence near delivery in this group. Notably, reaction strength among positive samples was predominantly low-grade (mostly 1+–2+). This pattern may reflect waning passive antibody levels crossing the assay’s detection threshold rather than a definitive biological difference between groups.

Maternal age was higher in twin than in singleton pregnancies, likely reflecting the increased use of assisted reproductive technology (ART) with advancing age [37, 38]. Despite being older on average, twin pregnancies showed a higher proportion of anti-D (indirect Coombs) negativity at delivery than singleton pregnancies, and this difference persisted after multivariable adjustment for maternal age, BMI, and the prophylaxis-to-testing interval (days) (Table 3). Maternal age was included primarily as an adjustment covariate and may act as a proxy for unmeasured factors—most notably ART status and related clinical differences—that were not available in this retrospective dataset. Some studies have reported associations between younger maternal age and RhD alloimmunization risk [39], and age-related differences in IgG kinetics have been described in other clinical contexts [40]; however, these observations are provided for context only and should not be interpreted as evidence of a causal age effect on anti-D detectability or as evidence regarding clinical protection. Accordingly, covariate estimates should be interpreted cautiously and not causally.

In twin pregnancies, the higher proportion of FMH risk compared with singleton pregnancies may be associated with an alloimmunisation risk that is greater than expected. Placental abruption and placenta previa, which are major causes of third-trimester bleeding, have been reported to occur more frequently in twin than in singleton pregnancies [41, 42]. Conversely, the tendency of twin pregnancies to deliver at earlier gestational ages than singleton pregnancies may contribute to the incidence of alloimmunisation being lower than anticipated [27, 28].

In our study, the proportion of women with undetectable maternal serum anti-D antibodies at delivery was higher in twin pregnancies than in singleton pregnancies.

Several strengths should be highlighted. We included a well-defined twin cohort, a population largely excluded from prior research on antenatal anti-D persistence, and used a contemporaneous singleton control group matched by completed gestational week at delivery, the primary determinant of detectability. In addition, antibody testing followed a standardized laboratory protocol on the same analyzer, thereby reducing measurement variability. A key strength is the use of a uniform antenatal prophylaxis protocol without dose adjustment by fetal number, reducing treatment variability. The twin–singleton comparative design with adjustment for key covariates provides novel real-world data on a pharmacokinetic surrogate outcome.

However, several limitations should be noted. First, the indirect Coombs test is qualitative (positive/negative); thus, detectability at delivery is a surrogate endpoint and does not necessarily reflect clinical protection against RhD alloimmunization. This is a major limitation because the assay does not provide an anti-D concentration or titer and may be sensitive to threshold effects. Second, selection bias is possible because inclusion required complete documentation and delivery within a single institution. Information bias/misclassification is also possible given the qualitative/semi-quantitative nature of the assay and potential variability in the exact timing of sampling at admission for delivery. This retrospective, single-center design limits causal inference and generalizability and cannot exclude residual confounding. Chorionicity and ART status were not routinely recorded in the retrospective dataset and therefore could not be analyzed or adjusted for; both may act as unmeasured confounders through differences in placental physiology and maternal characteristics. No formal sensitivity analyses were performed to assess the robustness of the findings under alternative model specifications. Unmeasured twin-related and clinical factors—including gestational weight gain, plasma volume expansion, surveillance intensity, hypertensive disorders, placenta previa/abruption, bleeding events, and institution-specific practice patterns—may have influenced antibody detectability. We did not assess clinical outcomes such as incident RhD alloimmunization or hemolytic disease of the fetus and newborn; therefore, clinical effectiveness cannot be inferred. Moreover, FMH was not quantified (e.g., by fetal cell screening), and we did not include potentially relevant factors such as mode of delivery or late-pregnancy interventions/events; therefore, unrecognized sensitizing events and residual bias cannot be fully excluded in this retrospective dataset. We could not account for several potential confounders that may influence distribution volume or antibody clearance, such as parity, obstetric complications, bleeding/sensitizing events, assisted reproduction, or peripartum interventions. Finally, without serial testing, the timing of loss of detectability is unknown and may have occurred at any point between the routine 28-week dose and delivery.

## Conclusion

A prospective study with predefined, serial anti-D antibody measurements at scheduled intervals after prophylaxis would provide more reliable data on the persistence of passive anti-D antibodies. Our findings are hypothesis-generating and suggest that passive anti-D may be less frequently detectable at delivery in twin pregnancies; however, clinical protection cannot be inferred from detectability at a single time point. Because we did not measure incident alloimmunization or neonatal outcomes (HDFN), any implication for dose modification in twin pregnancies remains purely hypothetical. Therefore, changes to current clinical practice cannot be recommended on the basis of our data alone. Future prospective studies should evaluate optimal prophylaxis strategies in twin pregnancies using serial measurements and clinical endpoints.

## Supplementary Information


Supplementary Material 1.


## Data Availability

The data that support the findings of this study are available from the corresponding author upon reasonable request.
