# Supplementary material for: Persistence of antenatal anti-D immunoglobulin G at delivery in twin versus singleton pregnancies: a retrospective cohort study
Source: BMC Pregnancy Childbirth. 2026 Jun 20;26:977. doi: 10.1186/s12884-026-09530-2 (PMC13531754; doi:10.1186/s12884-026-09530-2)
Supplement: Supplementary file 1 [file 12884_2026_9530_MOESM1_ESM.docx]

**Supplementary Table S1**

**Supplementary Table S1.** Distribution of indirect antiglobulin test (IAT) reaction grades at delivery in twin and singleton pregnancies.

| **IAT reaction grade** | **Twin pregnancies (n=21)** | **Singleton pregnancies (n=77)** |
| --- | --- | --- |
| **1+** | 9 (42.9%) | 27 (35.1%) |
| **2+** | 9 (42.9%) | 30 (39.0%) |
| **3+** | 3 (14.3%) | 19 (24.7%) |
| **4+** | 0 (0.0%) | 1 (1.3%) |

**Footnote**
Values are n (%). Grades are reported only for Coombs-positive samples (≥1+). *Descriptive distribution; no formal between-group comparison was performed due to sparse cells.*
